# Supplementary material for: A Biopsy-Controlled Prospective Study of Contrast-Enhancing Diffuse Glioma Infiltration Based on FET-PET and FLAIR
Source: Cancers (Basel). 2024 Mar 24;16(7):1265. doi: 10.3390/cancers16071265 (PMC11010945; doi:10.3390/cancers16071265)
Supplement: Supplementary file 1 [file cancers-16-01265-s001.zip › cancers-2857285-supplementary.pdf]

Supplementary Materials: A biopsy- controlled prospective study of contrast-enhancing diffuse glioma infiltration based on FET-PET and FLAIR.

Maciej Harat, Izabela Miechowicz, Józefina Rakowska, Izabela Zarębska and Bogdan Małkowski

Table S1. Summary of differences between PET parameters and trajectories at both timepoints.

Parameter	Overall		T1-GAD (A)		PET(B)		PET-(C)		FLAIR (D)			
Timepoint	10	60	10	60	10	60	10	60	10	60	Time 10 Difference between trajectory	Time 60 Difference between trajectory
SUV	1.6	1.8	2.3	2.6	1.9	2.1	0.9	1.1	1.1	1.4	A vs B p=0.089<br>A vs C p<0.001<br>A vs D p<0.001<br>B vs C p<0.001<br>B vs D p<0.001<br>C vs D p=0.709	A vs B p=0.025<br>A vs C p<0.001<br>A vs D p<0.001<br>B vs C p<0.001<br>B vs D p<0.001<br>C vs D p=0.709
Target to Brain	2.9	2.7	3.5	3.1	3.3	2.9	1.7	1.6	1.9	2.0	A vs B p=0.271<br>A vs C p<0.001<br>A vs D p<0.001<br>B vs C p<0.001<br>B vs D p<0.001<br>C vs D p<1.000	A vs B p=0.192<br>A vs C p<0.001<br>A vs D p<0.001<br>B vs C p<0.001<br>B vs D p<0.001<br>C vs D p=0.683
Target to plexus	1.6	1.9	2.0	2.4	1.8	2.0	0.8	1.2	0.9	1.4	A vs B p=0.498<br>A vs C p<0.001<br>A vs D p<0.001<br>B vs C p<0.001<br>B vs D p<0.001<br>C vs D p<1.000	A vs B p=0.143<br>A vs C p<0.001<br>A vs D p<0.001<br>B vs C p<0.001<br>B vs D p<0.001<br>C vs D p<1.000

Table S2 (a-d). Detailed values of SUV10 and SUV60 according to biopsy site.

a) T1-GAD+

Parameter	N	Mean	Median	Min	Max	Lower Q	Upper Q	SD
SUV10	91	2.40	2.27	0.79	4.47	1.51	3.26	1.01
SUV60	91	2.60	2.55	1.07	5.86	1.72	3.17	1.12

b) PET+

Parameter	N	Mean	Median	Min	Max	Lower Q	Upper Q	SD
SUV10	110	1.95	1.91	1.34	3.72	1.47	2.49	0.72
SUV60	110	2.11	2.15	1.56	5.03	1.57	2.60	0.85

c) FLAIR+

Parameter	N	Mean	Median	Min	Max	Lower Q	Upper Q	SD
SUV10	70	1.19	1.07	0.35	2.98	0.82	1.35	0.58
SUV60	71	1.47	1.37	0.43	4.09	0.99	1.72	0.76

d) PET-

Parameter	N	Mean	Median	Min	Max	Lower Q	Upper Q	SD
SUV10	11	0.84	0.87	0.58	1.05	0.66	0.91	0.16
SUV60	11	1.19	1.12	0.82	1.47	1.05	1.47	0.21

Table S2 B. p- values between various biopsy sites according to SUV 10 and SUV 60.

Parameter: SUV10	T1-GAD+ R:185.24	PET + R:157.07	FLAIR R:76.829	PET - R:35.500
T1-GAD+		0.088612	0.000000	0.000000
PET +	0.088612		0.000000	0.000015
FLAIR	0.000000	0.000000		0.708991
PET -	0.000000	0.000015	0.708991	
Parameter: SUV60	T1-GAD+ R:184.71	PET + R:151.49	FLAIR R:85.838	PET - R:56.273
T1-GAD+		0.025002	0.000000	0.000005
PET +	0.025002		0.000001	0.001405
FLAIR	0.000000	0.000001		1.000000
PET -	0.000005	0.001405	1.000000	

Figure S1. Plot showing SUV10 and SUV60 for different biospy trajcetories (biopsy sites).

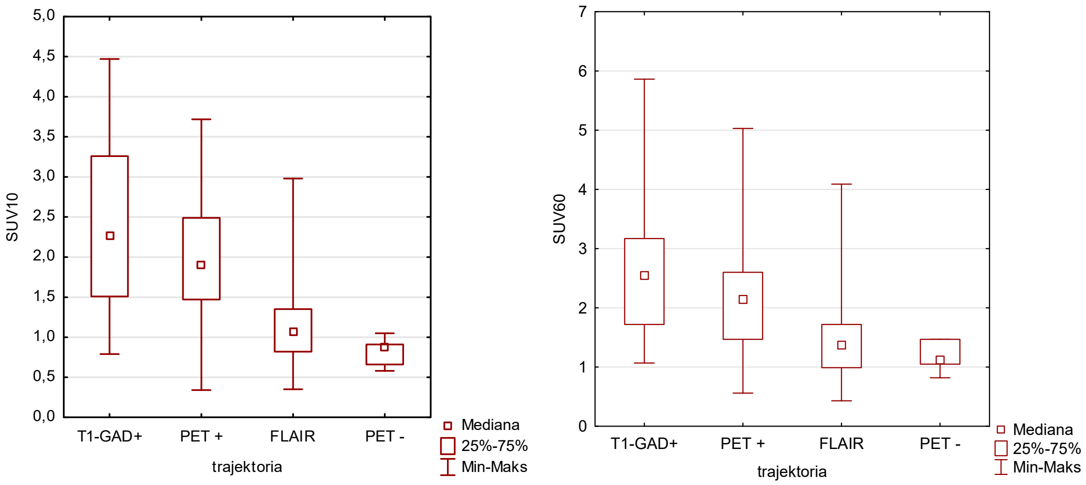

Table S2 (e-i). TBR values at both time points (10 and 60 minutes) using different reference anatomical structures overall (a) and related to trajectory (b-e)

e) overall

Parameter	N	Mean	Median	Min	Max	Lower Q	Upper Q	SD
SUV10/10 plexus	282	1.64	1.58	0.50	3.90	1.06	2.07	0.70
SUV10/10 thalamus max	282	1.90	1.61	0.49	5.00	1.25	2.38	1.00
SUV10/10 thalamus	282	2.46	2.11	0.59	6.77	1.54	3.15	1.29
SUV10/10 brain	282	3.08	2.90	0.94	7.45	2.05	3.78	1.36
SUV10/10 brain max	282	1.74	1.57	0.48	4.58	1.05	2.22	0.87
SUV10/10 middle A	282	2.44	2.15	0.65	6.25	1.55	3.18	1.22
SUV10/10 sinus	282	1.06	0.94	0.28	2.56	0.61	1.42	0.56
SUV60/60 sinus	283	1.49	1.31	0.45	3.73	0.95	1.76	0.73
SUV60/60 plexus	282	2.03	1.95	0.68	4.86	1.41	2.52	0.79
SUV60/60 thalamus max	275	1.75	1.60	0.56	4.80	1.26	2.12	0.78
SUV60/60 thalamus	283	2.27	2.10	0.82	6.10	1.59	2.76	0.97
SUV60/60 brain	283	2.77	2.73	0.93	6.34	1.95	3.36	1.09
SUV60/60 brain max	283	1.71	1.63	0.55	4.40	1.14	2.07	0.76
SUV60/60 middle A	283	2.38	2.22	0.66	5.28	1.67	3.02	1.00

f) T1-GAD +

Parameter	N	Mean	Median	Min	Max	Lower Q	Upper Q	SD
SUV10/10 plexus	91	2.03	1.96	0.97	3.90	1.44	2.35	0.70
SUV10/10 thalamus max	91	2.47	2.03	0.84	5.00	1.51	3.41	1.14
SUV10/10 thalamus	91	3.20	3.02	1.18	6.77	2.01	4.30	1.45
SUV10/10 brain	91	3.91	3.52	1.68	7.45	2.55	5.19	1.47
SUV10/10 brain max	91	2.19	2.08	0.75	4.58	1.45	2.88	1.00
SUV10/10 middle A	91	3.15	2.87	1.08	6.25	2.02	3.93	1.41
SUV10/10 sinus	91	1.38	1.35	0.41	2.56	0.80	1.96	0.61
SUV60/60 sinus	91	1.89	1.70	0.60	3.73	1.29	2.49	0.81
SUV60/60 plexus	91	2.48	2.35	1.13	4.86	1.92	2.94	0.85
SUV60/60 thalamus max	88	2.18	1.99	0.94	4.80	1.48	2.50	0.92
SUV60/60 thalamus	91	2.80	2.46	1.35	6.10	1.98	3.29	1.15
SUV60/60 brain	91	3.38	3.13	1.73	6.34	2.61	3.96	1.12
SUV60/60 brain max	91	2.10	1.96	0.99	4.40	1.52	2.48	0.83
SUV60/60 middle A	91	2.96	2.87	1.08	5.28	2.19	3.64	1.07

g) PET +

Parameter	N	Mean	Median	Min	Max	Lower Q	Upper Q	SD
SUV10/10 plexus	110	1.79	1.81	0.71	3.10	1.38	2.11	0.55
SUV10/10 thalamus max	110	1.99	1.66	0.74	4.29	1.47	2.36	0.79
SUV10/10 thalamus	110	2.59	2.31	0.97	5.81	1.84	3.15	1.01
SUV10/10 brain	110	3.29	3.30	1.03	6.20	2.60	3.83	0.96

Parameter	N	Mean	Median	Min	Max	Lower Q	Upper Q	SD
SUV10/10 brain max	110	1.86	1.67	0.72	3.93	1.41	2.23	0.69
SUV10/10 middle A	110	2.57	2.51	0.81	5.37	1.94	3.22	0.92
SUV10/10 sinus	110	1.10	1.07	0.33	2.45	0.76	1.39	0.45
SUV60/60 sinus	110	1.52	1.38	0.48	3.45	1.13	1.75	0.63
SUV60/60 plexus	110	2.15	2.03	1.00	3.41	1.79	2.53	0.61
SUV60/60 thalamus max	107	1.80	1.82	0.76	3.24	1.38	2.11	0.55
SUV60/60 thalamus	110	2.38	2.37	1.06	4.15	1.88	2.88	0.68
SUV60/60 brain	110	2.91	2.90	1.22	4.92	2.27	3.38	0.87
SUV60/60 brain max	110	1.80	1.74	0.76	3.36	1.31	2.20	0.62
SUV60/60 middle A	110	2.48	2.46	1.10	4.41	1.95	3.00	0.73

h) FLAIR

Parameter	N	Mean	Median	Min	Max	Lower Q	Upper Q	SD
SUV10/10 plexus	70	1.03	0.94	0.50	2.71	0.77	1.14	0.37
SUV10/10 thalamus max	70	1.19	1.18	0.52	3.20	0.83	1.39	0.47
SUV10/10 thalamus	70	1.52	1.43	0.71	4.08	1.06	1.72	0.62
SUV10/10 brain	70	1.92	1.86	1.06	4.89	1.45	2.19	0.69
SUV10/10 brain max	70	1.11	1.03	0.49	2.84	0.79	1.39	0.45
SUV10/10 middle A	70	1.50	1.51	0.65	3.31	1.14	1.83	0.54
SUV10/10 sinus	70	0.66	0.57	0.28	1.66	0.44	0.78	0.33
SUV60/60 sinus	71	1.02	0.97	0.46	2.42	0.69	1.21	0.43
SUV60/60 plexus	70	1.39	1.36	0.68	2.58	1.04	1.57	0.47
SUV60/60 thalamus max	69	1.25	1.29	0.57	2.46	0.76	1.67	0.51
SUV60/60 thalamus	71	1.60	1.57	0.82	3.03	1.03	2.02	0.58
SUV60/60 brain	71	1.95	1.95	0.93	3.70	1.28	2.61	0.74
SUV60/60 brain max	71	1.22	1.13	0.56	2.07	0.73	1.61	0.49

i) PET -

Parameter	N	Mean	Median	Min	Max	Lower Q	Upper Q	SD
SUV10/10 plexus	11	0.80	0.81	0.57	1.04	0.61	0.89	0.16
SUV10/10 thalamus max	11	0.77	0.88	0.49	0.92	0.67	0.88	0.16
SUV10/10 thalamus	11	0.98	1.07	0.59	1.21	0.88	1.16	0.22
SUV10/10 brain	11	1.54	1.69	0.94	1.90	1.38	1.81	0.34
SUV10/10 brain max	11	0.74	0.81	0.48	0.88	0.61	0.84	0.14
SUV10/10 middle A	11	1.23	1.18	0.86	1.67	0.98	1.43	0.28
SUV10/10 sinus	11	0.44	0.40	0.29	0.63	0.37	0.54	0.11
SUV60/60 sinus	11	0.89	0.94	0.45	1.31	0.61	1.31	0.32
SUV60/60 plexus	11	1.31	1.22	0.83	1.71	1.13	1.71	0.28
SUV60/60 thalamus max	11	0.89	0.83	0.56	1.17	0.77	1.17	0.20
SUV60/60 thalamus	11	1.22	1.13	0.83	1.52	1.08	1.52	0.22
SUV60/60 brain	11	1.52	1.62	1.19	1.75	1.21	1.69	0.24
SUV60/60 brain max	11	0.79	0.76	0.55	0.96	0.69	0.96	0.13
SUV60/60 middle A	11	1.12	1.05	0.76	1.40	1.00	1.40	0.20

Parameter: SUV10/10 plexus	T1-GAD+ R:185.86	PET + R:165.82	FLAIR R:62.950	PET - R:31.182
T1-GAD+		0.497851	0.000000	0.000000
PET +	0.497851		0.000000	0.000001
FLAIR	0.000000	0.000000		1.000000
PET -	0.000000	0.000001	1.000000	
Parameter: SUV60/60 plexus	T1-GAD+ R:184.64	PET + R:158.53	FLAIR R:72.614	PET - R:52.682
T1-GAD+		0.142960	0.000000	0.000002
PET +	0.142960		0.000000	0.000243
FLAIR	0.000000	0.000000		1.000000
PET -	0.000002	0.000243	1.000000	

Parameter: SUV10/10 thalamus max	T1-GAD+ R:185.51	PET + R:159.41	FLAIR R:74.736	PET - R:23.182
T1-GAD+		0.143727	0.000000	0.000000
PET +	0.143727		0.000000	0.000001
FLAIR	0.000000	0.000000		0.307691
PET -	0.000000	0.000001	0.307691	
Parameter: SUV60/60 thalamus max	T1-GAD+ R:176.90	PET + R:150.57	FLAIR R:85.326	PET - R:34.955
T1-GAD+		0.128257	0.000000	0.000000
PET +	0.128257		0.000001	0.000026
FLAIR	0.000000	0.000001		0.306427
PET -	0.000000	0.000026	0.306427	

Parameter: SUV10/10 thalamus	T1-GAD+ R:186.63	PET + R:160.50	FLAIR R:72.007	PET - R:20.364
T1-GAD+		0.142759	0.000000	0.000000
PET +	0.142759		0.000000	0.000000
FLAIR	0.000000	0.000000		0.305272
PET -	0.000000	0.000000	0.305272	
Parameter: SUV60/60 thalamus	T1-GAD+ R:180.96	PET + R:161.54	FLAIR R:78.162	PET - R:36.409
T1-GAD+		0.564130	0.000000	0.000000
PET +	0.564130		0.000000	0.000008
FLAIR	0.000000	0.000000		0.692228
PET -	0.000000	0.000008	0.692228	

Parameter: SUV10/10 brain	T1-GAD+ R:187.05	PET + R:163.91	FLAIR R:63.921	PET - R:34.182
T1-GAD+		0.271364	0.000000	0.000000

Parameter: SUV10/10 brain	T1-GAD+ R:187.05	PET + R:163.91	FLAIR R:63.921	PET - R:34.182
PET +	0.271364		0.000000	0.000003
FLAIR	0.000000	0.000000		1.000000
PET -	0.000000	0.000003	1.000000	
Parameter: SUV60/60 brain	T1-GAD+ R:183.81	PET + R:158.93	FLAIR R:78.514	PET - R:36.591
T1-GAD+		0.191697	0.000000	0.000000
PET +	0.191697		0.000000	0.000014
FLAIR	0.000000	0.000000		0.683372
PET -	0.000000	0.000014	0.683372	

Parameter: SUV10/10 brain max	T1-GAD+ R:180.43	PET + R:162.25	FLAIR R:76.321	PET - R:26.727
T1-GAD+		0.694198	0.000000	0.000000
PET +	0.694198		0.000000	0.000001
FLAIR	0.000000	0.000000		0.364739
PET -	0.000000	0.000001	0.364739	
Parameter: SUV60/60 brain max	T1-GAD+ R:181.10	PET + R:157.37	FLAIR R:85.887	PET - R:26.955
T1-GAD+		0.244316	0.000000	0.000000
PET +	0.244316		0.000000	0.000003
FLAIR	0.000000	0.000000		0.157555
PET -	0.000000	0.000003	0.157555	

Parameter: SUV10/10 middle A	T1-GAD+ R:184.68	PET + R:160.98	FLAIR R:70.386	PET - R:42.000
T1-GAD+		0.241697	0.000000	0.000000
PET +	0.241697		0.000000	0.000024
FLAIR	0.000000	0.000000		1.000000
PET -	0.000000	0.000024	1.000000	
Parameter: SUV60/60 middle A	T1-GAD+ R:187.34	PET + R:155.26	FLAIR R:80.556	PET - R:30.864
T1-GAD+		0.034050	0.000000	0.000000
PET +	0.034050		0.000000	0.000009
FLAIR	0.000000	0.000000		0.365654
PET -	0.000000	0.000009	0.365654	

Parameter: SUV10/10 sinus	T1-GAD+ R:184.26	PET + R:155.43	FLAIR R:80.457	PET - R:36.909
T1-GAD+		0.075497	0.000000	0.000000

Parameter: SUV10/10 sinus	T1-GAD+ R:184.26	PET + R:155.43	FLAIR R:80.457	PET - R:36.909
PET +	0.075497		0.000000	0.000026
FLAIR	0.000000	0.000000		0.598046
PET -	0.000000	0.000026	0.598046	
Parameter: SUV60/60 sinus	T1-GAD+ R:186.54	PET + R:151.74	FLAIR R:81.430	PET - R:67.045
T1-GAD+		0.016143	0.000000	0.000029
PET +	0.016143		0.000000	0.006393
FLAIR	0.000000	0.000000		1.000000
PET -	0.000029	0.006393	1.000000	

Tables S3. Detailed values of PET parameters between tumor, astrogliosis, and normal brain stuctures.

SUV 10 and SUV 60 specific to tumor and astrogliosis:

Overall	N	Mean	Median	Min	Max	Lower Q	Upper Q	SD
SUV10	282	1.86	1.63	0.34	4.47	1.18	2.52	0.93
SUV60	283	2.07	1.79	0.43	5.86	1.33	2.61	1.02
Astrogliosis	N	Mean	Median	Min	Max	Lower Q	Upper Q	SD
SUV10	52	1.12	1.04	0.34	2.98	0.76	1.47	0.54
SUV60	53	1.40	1.21	0.43	2.61	1.02	1.83	0.57
Tumor	N	Mean	Median	Min	Max	Lower Q	Upper Q	SD
SUV10	230	2.03	1.88	0.50	4.47	1.35	2.66	0.92
SUV60	230	2.22	2.08	0.53	5.86	1.46	2.73	1.03

Differences in SUV values between astrogliosis and various grades

Parameter: SUV10	astrogliosis R:71.760	G2 R:93.844	G3 R:160.70	G4 R:197.68
astrogliosis		1.000000	0.000000	0.000000
G2	1.000000		0.000014	0.000000
G3	0.000000	0.000014		0.023370
G4	0.000000	0.000000	0.023370	

Parameter: SUV60	astrogliosis R:83.972	G2 R:102.58	G3 R:161.04	G4 R:183.15
astrogliosis		1.000000	0.000000	0.000000
G2	1.000000		0.000238	0.000004
G3	0.000000	0.000238		0.512747
G4	0.000000	0.000004	0.512747	

Astrogliosis uptake (SUV10 and SUV 60) vs other structures

Parameter	N	Mean	Median	Min	Max	Lower Q	Upper Q	SD
SUV10	52	1.12	1.04	0.34	2.98	0.76	1.47	0.54
SUV PLEXUS 10	54	1.10	1.12	0.48	1.68	0.85	1.40	0.34
THALAMUS MAX 10	54	0.87	0.93	0.46	1.13	0.84	0.99	0.17
THALAMUS 10	54	0.71	0.75	0.35	0.86	0.67	0.81	0.15
BRAIN 10	54	0.54	0.58	0.33	0.70	0.47	0.61	0.11
BRAIN 10 MAX	54	1.03	1.05	0.47	1.50	1.01	1.08	0.25
Middle A. 10	54	0.76	0.77	0.42	1.03	0.73	0.89	0.16
SINUS 10	54	2.01	2.05	0.71	3.25	1.80	2.28	0.62
Parameter	N	Mean	Median	Min	Max	Lower Q	Upper Q	SD
SUV60	53	1.40	1.21	0.43	2.61	1.02	1.83	0.57
SINUS 60	54	1.49	1.52	0.82	2.08	1.01	1.84	0.43
SUV PLEXUS 60	54	1.04	1.01	0.56	1.37	0.86	1.31	0.27
THALAMUS Max 60	54	1.16	1.11	0.66	1.81	1.03	1.46	0.28
THALAMUS 60	54	0.89	0.99	0.43	1.29	0.76	1.05	0.22
BRAIN 60	54	0.72	0.75	0.45	0.97	0.62	0.82	0.15
BRAIN 60 MAX	54	1.18	1.23	0.60	1.48	1.00	1.44	0.28
MIDDLE A. 60	54	0.90	0.82	0.51	1.31	0.71	1.08	0.26

Comparison of SUV between astrogliosis and various anatomical structures

**p-value**	**SUV10**	**SUV PLEXUS 10**	**THALAMUS MAX 10**	**THALAMUS 10**	**BRAIN 10**	**BRAIN 10 MAX**	**Middle A. 10**	**SINUS 10**
SUV10		0.509097	1	0.000008	<0.000001	1	0.007548	<0.000001
SUV PLEXUS 10	0.509097		0.018681	<0.000001	<0.000001	1	<0.000001	0.013894
THALAMUS MAX 10	1	0.018681		0.001141	<0.000001	0.151164	0.259447	<0.000001
THALAMUS 10	0.000008	<0.000001	0.001141		0.080083	<0.000001	1	<0.000001
BRAIN 10	<0.000001	<0.000001	<0.000001	0.080083		<0.000001	0.000206	<0.000001
BRAIN 10 MAX	1	1	0.151164	<0.000001	<0.000001		0.000002	0.001141
Middle A. 10	0.007548	<0.000001	0.259447	1	0.000206	0.000002		<0.000001
SINUS 10	<0.000001	0.013894	<0.000001	<0.000001	<0.000001	0.001141	<0.000001	
**p value**	SUV60	SINUS 60	SUV PLEXUS 60	THALAMUS Max 60	THALAMUS 60	BRAIN 60	BRAIN 60 MAX	MIDDLE A. 60
SUV60		1	0.013554	1	<0.000001	<0.000001	1	<0.000001
SINUS 60	1		0.000011	0.666653	<0.000001	<0.000001	0.777168	<0.000001
SUV PLEXUS 60	0.013554	0.000011		0.136435	0.026031	<0.000001	0.113195	0.485867
THALAMUS Max 60	1	0.666653	0.136435		<0.000001	<0.000001	1	0.000006
THALAMUS 60	<0.000001	<0.000001	0.026031	<0.000001		0.113195	<0.000001	1
BRAIN 60	<0.000001	<0.000001	<0.000001	<0.000001	0.113195		<0.000001	0.003944
BRAIN 60 MAX	1	0.777168	0.113195	1	<0.000001	<0.000001		0.000004
MIDDLE A. 60	<0.000001	<0.000001	0.485867	0.000006	1	0.003944	0.000004	

Comparison of tumor uptake (SUV10 and SUV 60) vs other structures

The mean SUV in tumor of any grade at 10 min a.r.i. was 2.0, similar to the SUV in the sinus (1.8) and significantly higher than the SUV in other anatomical structures.

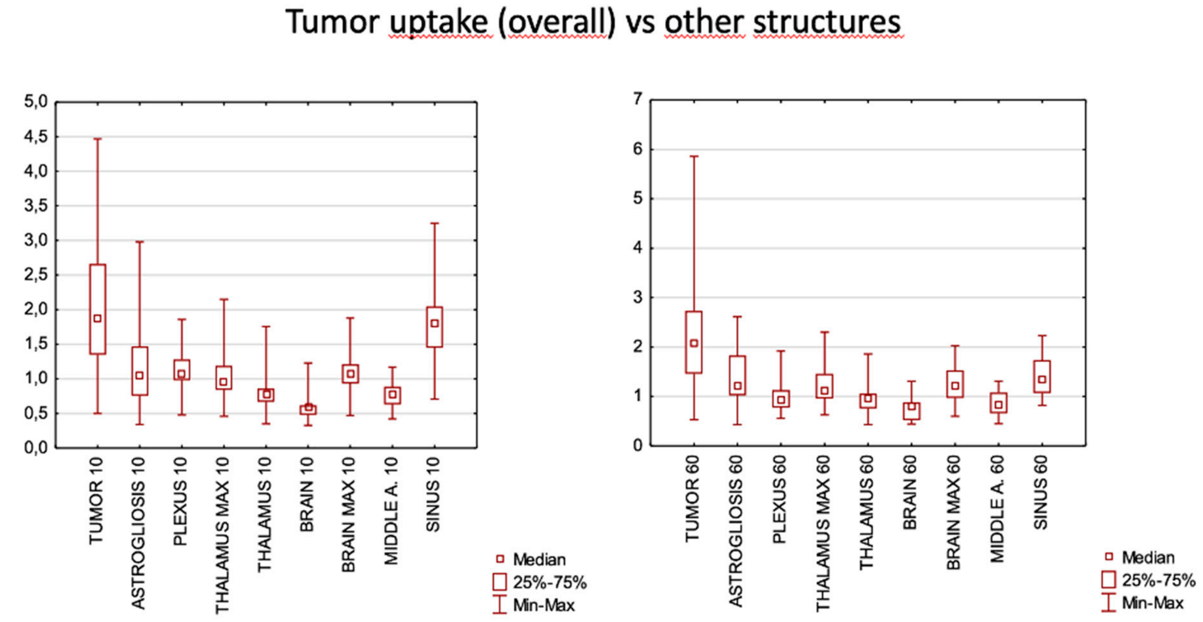

Parameter	N	Mean	Median	Min	Max	Lower Q
SUV10	230	2.03	1.88	0.50	4.47	1.35
SUV PLEXUS 10	230	1.13	1.07	0.48	1.86	0.98
THALAMUS MAX 10	230	1.06	0.97	0.46	2.15	0.84
THALAMUS	230	0.81	0.77	0.35	1.76	0.67
BRAIN 10	230	0.62	0.60	0.33	1.23	0.48
BRAIN 10 MAX	230	1.11	1.08	0.47	1.88	0.93
Middle A. 10	230	0.78	0.77	0.42	1.17	0.63

Parameter	N	Mean	Median	Min	Max	Lower Q	Upper Q	SD
SINUS 10	230	1.82	1.80	0.71	3.25	1.45		
SUV10							2.66	0.92

Parameter	N	Mean	Median	Min	Max	Lower Q	Upper Q	SD
SUV60	222	2.21	2.05	0.53	5.86	1.46	2.68	1.04
SINUS 60	222	1.43	1.45	0.82	2.23	1.07	1.74	0.39
SUV PLEXUS 60	221	1.01	0.91	0.56	1.92	0.77	1.26	0.32
THALAMUS Max 60	222	1.22	1.12	0.63	2.3	0.96	1.46	0.41
THALAMUS 60	222	0.93	0.97	0.43	1.86	0.76	1.06	0.31
BRAIN 60	222	0.75	0.76	0.44	1.31	0.52	0.87	0.23
BRAIN 60 MAX	222	1.24	1.23	0.60	2.03	0.97	1.53	0.37
MIDDLE A. 60	222	0.88	0.83	0.45	1.31	0.66	1.05	0.25

**p value**	**SUV10**	**SUV PLEXUS 10**	**THALAMUS MAX 10**	**THALAMUS**	**BRAIN 10**	**BRAIN 10 MAX**	**Middle A. 10**	**SINUS 10**
SUV10		<0.000001	<0.000001	<0.000001	<0.000001	<0.000001	<0.000001	1
SUV PLEXUS 10	<0.000001		1	<0.000001	<0.000001	1	<0.000001	<0.000001
THALAMUS MAX 10	<0.000001	1		<0.000001	<0.000001	0.820309	<0.000001	<0.000001
THALAMUS	<0.000001	<0.000001	<0.000001		0.000007	<0.000001	1	<0.000001
BRAIN 10	<0.000001	<0.000001	<0.000001	0.000007		<0.000001	<0.000001	<0.000001
BRAIN 10 MAX	<0.000001	1	0.820309	<0.000001	<0.000001		<0.000001	<0.000001
Middle A. 10	<0.000001	<0.000001	<0.000001	1	<0.000001	<0.000001		<0.000001
SINUS 10	1	<0.000001	<0.000001	<0.000001	<0.000001	<0.000001	<0.000001	
**p value**	SUV60	SINUS 60	SUV PLEXUS 60	THALAMUS Max 60	THALAMUS 60	BRAIN 60	BRAIN 60 MAX	MIDDLE A. 60
SUV60		<0.000001	<0.000001	<0.000001	<0.000001	<0.000001	<0.000001	<0.000001
SINUS 60	<0.000001		<0.000001	1	<0.000001	<0.000001	1	<0.000001
SUV PLEXUS 60	<0.000001	<0.000001		<0.000001	0.206303	<0.000001	<0.000001	0.000029
THALAMUS Max 60	<0.000001	1	<0.000001		<0.000001	<0.000001	1	<0.000001
THALAMUS 60	<0.000001	<0.000001	0.206303	<0.000001		<0.000001	<0.000001	0.770707
BRAIN 60	<0.000001	<0.000001	<0.000001	<0.000001	<0.000001		<0.000001	0.000013
BRAIN 60 MAX	<0.000001	1	<0.000001	1	<0.000001	<0.000001		<0.000001
MIDDLE A. 60	<0.000001	<0.000001	0.000029	<0.000001	0.770707	0.000013	<0.000001	

In grade 2 tumors, the tumor SUV_{10} (1.35) was not significantly different to astrogliosis (1.1), plexus (1.0), brain max (1.1), or sinus 10 (1.8) but significantly higher than the other analyzed structures. The SUV was 2.1 in grade 3 tumors and 2.5 in grade 4 tumors, significantly higher than in all analyzed structures but sinus. The mean SUV in tumor (2.2) was significantly different to all other anatomical structures at 60 min a.r.i. In grade 2 tumors, the SUV_{60} was 1.6, similar to astrogliosis (1.4), sinus (1.4), thalamus max (1.2), and brain max (1.3) but significantly higher than in other analyzed structures including choroid plexus 60 (0.98). The SUV was 2.1 in grade 3 samples and 2.5 in grade 4 samples, significantly higher than in all analyzed structures but sinus.

There was significantly higher uptake in astrogliosis than in brain 10 and 60 minutes a.r.i., similar to choroid plexus 10 minutes a.r.i. (1.12 vs 1.12; p=0.5) but significantly higher than plexus 60 (p=0.013).

Grade 2 (SUV10 and SUV 60) vs other structures

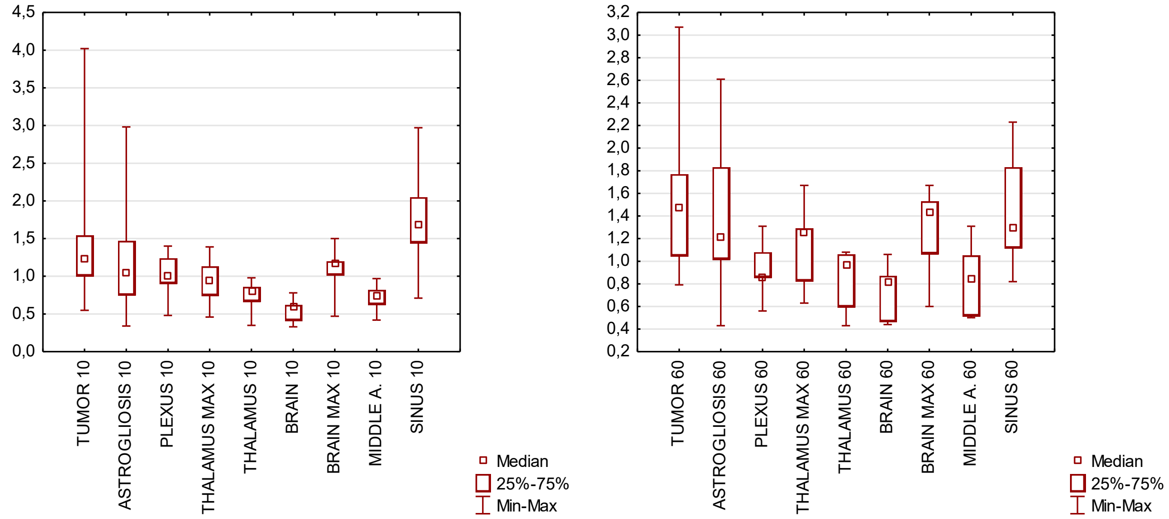

Grade 3 (SUV10 and SUV 60) vs other structures

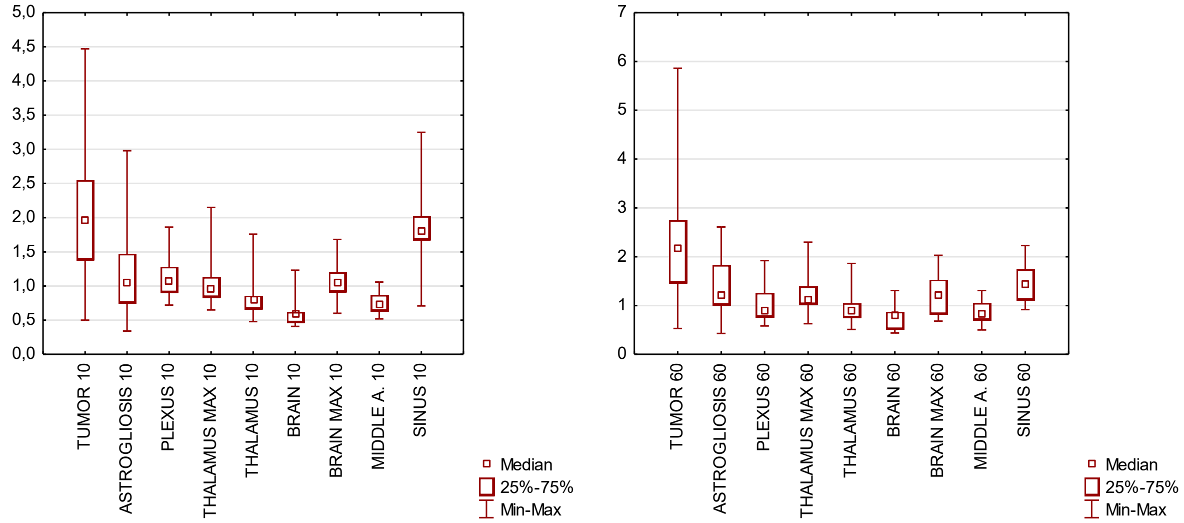

Grade 4 (SUV10 and SUV 60) vs other structures

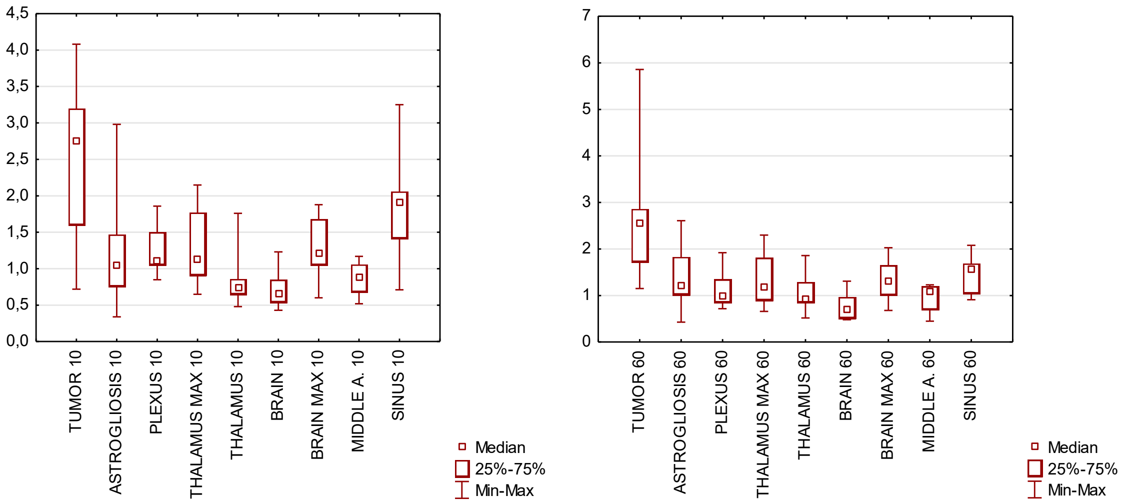

Grade 2 (SUV10 and SUV 60) vs other structures

Parameter	N	Mean	Median	Min	Max	Lower Q
SUV10	45	1.35	1.23	0.55	4.02	1.01
SUV PLEXUS 10	45	1.02	1.01	0.48	1.40	0.91
THALAMUS MAX 10	45	0.92	0.94	0.46	1.39	0.75
THALAMUS	45	0.74	0.81	0.35	0.98	0.67
BRAIN 10	45	0.55	0.60	0.33	0.78	0.42
BRAIN 10 MAX	45	1.06	1.17	0.47	1.50	1.02
Middle A. 10	45	0.73	0.73	0.42	0.97	0.63
SINUS 10	45	1.77	1.68	0.71	2.97	1.45

Parameter	Upper Q	SD
SUV10	1.54	0.60
SUV PLEXUS 10	1.24	0.25
THALAMUS MAX 10	1.13	0.23
THALAMUS	0.86	0.19
BRAIN 10	0.62	0.13
BRAIN 10 MAX	1.20	0.23
Middle A. 10	0.82	0.15
SINUS 10	2.05	0.50

Parameter	N	Mean	Median	Min	Max	Lower Q
SUV60	45	1.60	1.47	0.79	3.07	1.05
SINUS 60	45	1.40	1.30	0.82	2.23	1.12
SUV PLEXUS 60	44	0.93	0.86	0.56	1.31	0.86
THALAMUS Max 60	45	1.15	1.26	0.63	1.67	0.83
THALAMUS 60	45	0.86	0.97	0.43	1.08	0.60
BRAIN 60	45	0.75	0.82	0.44	1.06	0.47

Parameter	N	Mean	Median	Min	Max	Lower Q
BRAIN 60 MAX	45	1.30	1.44	0.60	1.67	1.07
MIDDLE A. 60	45	0.84	0.84	0.50	1.31	0.52

Parameter	Upper Q	SD
SUV60	1.77	0.62
SINUS 60	1.83	0.45
SUV PLEXUS 60	1.08	0.21
THALAMUS Max 60	1.29	0.30
THALAMUS 60	1.06	0.23
BRAIN 60	0.87	0.22
BRAIN 60 MAX	1.53	0.33
MIDDLE A. 60	1.05	0.26

**p value**	**SUV10**	**SUV PLEXUS 10**	**THALAMUS MAX 10**	**THALAMUS**	**BRAIN 10**	**BRAIN 10 MAX**	**Middle A. 10**	**SINUS 10**
SUV10		1	0.006548	<0.000001	<0.000001	1	<0.000001	0.126244
SUV PLEXUS 10	1		0.746965	<0.000001	<0.000001	1	0.000062	0.000471
THALAMUS MAX 10	0.006548	0.746965		0.007744	<0.000001	0.17594	0.330992	<0.000001
THALAMUS	<0.000001	<0.000001	0.007744		0.668316	<0.000001	1	<0.000001
BRAIN 10	<0.000001	<0.000001	<0.000001	0.668316		<0.000001	0.020437	<0.000001
BRAIN 10 MAX	1	1	0.17594	<0.000001	<0.000001		0.000004	0.004271
Middle A. 10	<0.000001	0.000062	0.330992	1	0.020437	0.000004		<0.000001
SINUS 10	0.126244	0.000471	<0.000001	<0.000001	<0.000001	0.004271	<0.000001	
**p value**	SUV60	SINUS 60	SUV PLEXUS 60	THALAMUS Max 60	THALAMUS 60	BRAIN 60	BRAIN 60 MAX	MIDDLE A. 60
SUV60		1	<0.000001	0.414571	<0.000001	<0.000001	1	<0.000001
SINUS 60	1		0.000002	1	<0.000001	<0.000001	1	<0.000001
SUV PLEXUS 60	<0.000001	0.000002		0.000749	1	0.020838	<0.000001	1
THALAMUS Max 60	0.414571	1	0.000749		0.000006	<0.000001	1	<0.000001
THALAMUS 60	<0.000001	<0.000001	1	0.000006		0.495636	<0.000001	1
BRAIN 60	<0.000001	<0.000001	0.020838	<0.000001	0.495636		<0.000001	1
BRAIN 60 MAX	1	1	<0.000001	1	<0.000001	<0.000001		<0.000001
MIDDLE A. 60	<0.000001	<0.000001	1	<0.000001	1	1	<0.000001	

Tables S4. Specific Target-to-Background Ratios (TBR) in tumor. astrogliosis and anatomical structures.

Astrogliosis

**Parameter**	**N**	**Mean**	**Median**	**Min**	**Max**	**Lower Q**	**Upper q**	**SD**
SUV10/10 plexus	52	0.98	0.90	0.59	2.05	0.77	1.10	0.31
SUV10/10 thalamus max	52	1.29	1.22	0.54	3.20	0.82	1.69	0.62
SUV10/10 thalamus	52	1.55	1.33	0.76	4.08	1.05	1.77	0.73
SUV10/10 brain	52	2.01	1.90	1.03	5.21	1.40	2.35	0.90
SUV10/10 brain max	52	1.10	0.94	0.49	2.84	0.73	1.41	0.53
SUV10/10 middle A	52	1.45	1.34	0.70	3.62	0.95	1.82	0.62
SUV10/10 sinus	52	0.57	0.51	0.28	1.42	0.38	0.70	0.28
SUV60/60 sinus	53	0.96	0.97	0.46	1.64	0.66	1.19	0.33
SUV60/60 plexus	53	1.33	1.27	0.68	2.58	1.02	1.57	0.45
SUV60/60 thalamus max	53	1.24	1.07	0.57	2.46	0.76	1.65	0.52
SUV60/60 thalamus	53	1.58	1.56	0.82	3.03	1.03	1.95	0.55

Parameter	N	Mean	Median	Min	Max	Lower Q	Upper q	SD
SUV60/60 brain	53	1.92	1.88	0.96	3.70	1.28	2.34	0.67
SUV60/60 brain max	53	1.20	1.13	0.60	2.07	0.73	1.50	0.45
SUV60/60 middle A	53	1.60	1.65	0.66	2.82	1.10	2.10	0.63

G3

Parameter	N	Mean	Median	Min	Max	Lower Q	Upper Q	SD
SUV10/10 plexus	125	1.85	1.77	0.50	3.90	1.37	2.17	0.73
SUV10/10 thalamus max	125	2.14	1.85	0.52	5.00	1.41	2.92	1.05
SUV10/10 thalamus	125	2.70	2.42	0.71	6.77	1.70	3.29	1.29
SUV10/10 brain	125	3.46	3.28	1.16	7.45	2.42	4.20	1.38
SUV10/10 brain max	125	2.00	1.81	0.60	4.58	1.39	2.52	0.90
SUV10/10 middle A	125	2.76	2.44	0.65	6.25	1.92	3.52	1.30
SUV10/10 sinus	125	1.15	1.07	0.28	2.35	0.80	1.50	0.52
SUV60/60 sinus	125	1.65	1.57	0.45	3.73	1.03	2.03	0.78
SUV60/60 plexus	125	2.31	2.13	0.70	4.86	1.82	2.90	0.85
SUV60/60 thalamus max	125	1.97	1.97	0.56	4.80	1.44	2.34	0.81
SUV60/60 thalamus	125	2.56	2.43	0.83	6.10	1.96	3.04	1.02
SUV60/60 brain	125	3.06	3.07	0.99	6.34	2.32	3.61	1.09
SUV60/60 brain max	125	1.94	1.96	0.55	4.40	1.45	2.31	0.78
SUV60/60 middle A	125	2.63	2.70	0.76	5.28	2.01	3.27	0.98

G2

Parameter	N	Mean	Median	Min	Max	Lower Q	Upper Q	SD
SUV10/10 plexus	45	1.37	1.15	0.57	3.14	0.96	1.63	0.55
SUV10/10 thalamus max	45	1.52	1.51	0.49	4.02	1.22	1.75	0.61
SUV10/10 thalamus	45	1.88	1.87	0.59	4.96	1.43	2.17	0.73
SUV10/10 brain	45	2.52	2.30	0.94	6.70	1.74	3.28	1.09
SUV10/10 brain max	45	1.30	1.29	0.48	3.44	0.95	1.48	0.50
SUV10/10 middle A	45	1.87	1.72	0.92	4.79	1.40	2.11	0.74
SUV10/10 sinus	45	0.81	0.73	0.35	2.45	0.58	0.87	0.42
SUV60/60 sinus	45	1.24	1.18	0.51	3.34	0.84	1.37	0.63
SUV60/60 plexus	44	1.70	1.75	0.88	2.83	1.33	1.96	0.45
SUV60/60 thalamus max	45	1.41	1.39	0.59	2.37	1.13	1.67	0.42
SUV60/60 thalamus	45	1.89	1.82	0.93	2.92	1.52	2.37	0.53
SUV60/60 brain	45	2.20	2.19	0.93	3.74	1.69	2.62	0.71
SUV60/60 brain max	45	1.26	1.24	0.59	2.13	0.96	1.54	0.40
SUV60/60 middle A	45	1.98	2.00	0.95	3.64	1.50	2.24	0.65

G4

Parameter	N	Mean	Median	Min	Max	Lower Q	Upper Q	SD
SUV10/10 plexus	60	1.97	2.00	0.53	2.99	1.70	2.33	0.51
SUV10/10 thalamus max	60	2.19	1.65	0.84	4.92	1.48	3.22	1.09
SUV10/10 thalamus	60	3.18	2.97	1.50	6.67	1.95	4.27	1.37

Parameter	N	Mean	Median	Min	Max	Lower Q	Upper Q	SD
SUV10/10 brain	60	3.63	3.41	1.31	5.82	2.79	4.81	1.17
SUV10/10 brain max	60	2.07	1.96	0.79	4.51	1.46	2.48	0.85
SUV10/10 middle A	60	3.04	2.89	1.26	6.15	2.34	3.69	1.05
SUV10/10 sinus	60	1.47	1.50	0.50	2.56	1.04	1.89	0.52
SUV60/60 sinus	60	1.82	1.68	0.99	3.47	1.30	2.18	0.65
SUV60/60 plexus	60	2.31	2.29	1.23	4.01	1.93	2.77	0.58
SUV60/60 thalamus max	52	2.02	1.85	0.93	4.80	1.46	2.37	0.79
SUV60/60 thalamus	60	2.57	2.33	1.30	6.10	2.02	2.94	0.99
SUV60/60 brain	60	3.33	3.28	1.52	6.34	2.65	3.95	1.00
SUV60/60 brain max	60	2.02	1.93	0.81	4.40	1.47	2.34	0.76
SUV60/60 middle A	60	2.84	2.50	1.12	5.28	2.15	3.54	1.04

Parameter:<br>SUV10/10 plexus	astrogliosis<br>R:56.788	G2<br>R:110.73	G3<br>R:164.76	G4<br>R:189.53
astrogliosis		0.006950	0.000000	0.000000
G2	0.006950		0.000830	0.000006
G3	0.000000	0.000830		0.319240
G4	0.000000	0.000006	0.319240	
Zależna:<br>SUV60/60 plexus	Wartość p dla porównań wielokrotnych (dwustronych); SUV60/60 plexus (DO STATYSTYKI_1)<br>Zmienna niezależna (grupująca): diagnoza<br>Test Kruskala-Wallisa: H (3. N= 282) =84.95388 p =.0000			
	astrogliosis<br>R:62.462	G2<br>R:106.73	G3<br>R:169.24	G4<br>R:179.03
astrogliosis		0.046688	0.000000	0.000000
G2	0.046688		0.000074	0.000048
G3	0.000000	0.000074		1.000000
G4	0.000000	0.000048	1.000000	

Parameter: SUV10/10<br>thalamus max	astrogliosis<br>R:85.750	G2<br>R:117.29	G3<br>R:161.14	G4<br>R:167.06
astrogliosis		0.344986	0.000000	0.000001
G2	0.344986		0.011886	0.011819
G3	0.000000	0.011886		1.000000
G4	0.000001	0.011819	1.000000	
Zależna:<br>SUV60/60 thalamus max	Wartość p dla porównań wielokrotnych (dwustronych); SUV60/60 thalamus max (DO STATYSTYKI_1)<br>Zmienna niezależna (grupująca): diagnoza<br>Test Kruskala-Wallisa: H (3. N= 275) =54.41983 p =.0000			
	astrogliosis<br>R:82.094	G2<br>R:101.74	G3<br>R:163.40	G4<br>R:165.30
astrogliosis		1.000000	0.000000	0.000000
G2	1.000000		0.000049	0.000521
G3	0.000000	0.000049		1.000000

Parameter: SUV10/10 thalamus max	astrogliosis R:85.750	G2 R:117.29	G3 R:161.14	G4 R:167.06
G4	0.000000	0.000521	1.000000	

Parameter: SUV10/10 thalamus	astrogliosis R:72.240	G2 R:108.31	G3 R:159.83	G4 R:188.22
astrogliosis		0.178930	0.000000	0.000000
G2	0.178930		0.001674	0.000004
G3	0.000000	0.001674		0.159816
G4	0.000000	0.000004	0.159816	
Parameter: SUV60/60 thalamus	astrogliosis R:75.604	G2 R:109.37	G3 R:168.80	G4 R:169.29
astrogliosis		0.250979	0.000000	0.000000
G2	0.250979		0.000177	0.001229
G3	0.000000	0.000177		1.000000
G4	0.000000	0.001229	1.000000	

Parameter: SUV10/10 brain	astrogliosis R:70.529	G2 R:107.68	G3 R:165.14	G4 R:179.13
astrogliosis		0.151566	0.000000	0.000000
G2	0.151566		0.000303	0.000053
G3	0.000000	0.000303		1.000000
G4	0.000000	0.000053	1.000000	
Parameter: SUV60/60 brain	astrogliosis R:73.660	G2 R:97.722	G3 R:166.07	G4 R:185.43
astrogliosis		0.881623	0.000000	0.000000
G2	0.881623		0.000009	0.000000
G3	0.000000	0.000009		0.792903
G4	0.000000	0.000000	0.792903	

Parameter: SUV10/10 brain max	astrogliosis R:73.346	G2 R:99.333	G3 R:168.18	G4 R:176.60
astrogliosis		0.705296	0.000000	0.000000
G2	0.705296		0.000007	0.000009
G3	0.000000	0.000007		1.000000
G4	0.000000	0.000009	1.000000	
Parameter: SUV60/60 brain max	astrogliosis R:81.708	G2 R:89.444	G3 R:170.61	G4 R:175.07
astrogliosis		1.000000	0.000000	0.000000
G2	1.000000		0.000000	0.000001
G3	0.000000	0.000000		1.000000
G4	0.000000	0.000001	1.000000	

Parameter: SUV10/10 middle A	astrogliosis R:65.250	G2 R:102.54	G3 R:163.38	G4 R:191.22
astrogliosis		0.148166	0.000000	0.000000
G2	0.148166		0.000107	0.000000
G3	0.000000	0.000107		0.178078
G4	0.000000	0.000000	0.178078	
Parameter: SUV60/60 middle A	astrogliosis R:75.764	G2 R:106.99	G3 R:166.09	G4 R:176.57
astrogliosis		0.358845	0.000000	0.000000
G2	0.358845		0.000196	0.000097
G3	0.000000	0.000196		1.000000
G4	0.000000	0.000097	1.000000	

Parameter: SUV10/10 sinus	astrogliosis R:62.519	G2 R:106.13	G3 R:158.71	G4 R:200.63
astrogliosis		0.051720	0.000000	0.000000
G2	0.051720		0.001251	0.000000
G3	0.000000	0.001251		0.006391
G4	0.000000	0.000000	0.006391	
Parameter: SUV60/60 sinus	astrogliosis R:74.717	G2 R:108.94	G3 R:160.26	G4 R:188.19
astrogliosis		0.234554	0.000000	0.000000
G2	0.234554		0.001862	0.000005
G3	0.000000	0.001862		0.178496
G4	0.000000	0.000005	0.178496	

Figure S2. Differentiation of tumor from astrogliosis based on target-to-brain ratios within FLAIR and outside CE+ and PET+.

Figure S2a

Differentiation of tumor from astrogliosis based on target-to-brain ratio within FLAIR

STANDARD AQUISITION (PET 60) TBR **>=1,6** SUV uptake in normal brain (VOI mean)

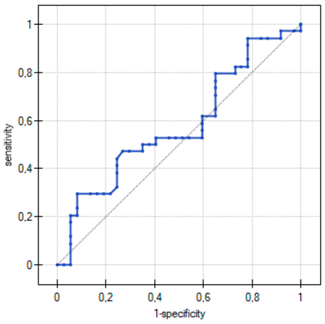

TBR brain	TBR60<1,6	TBR60>/=1,6	Suma
True astrogliosis	15	21	36
True tumor	11	23	34
**Sum**	**26**	**44**	**70**

**sensitivity**	**specificity**	**PPV**	**NPV**	**AUC (95%CI)**	**p**
0,56	0,41	0,46	0,50	0,579 (0,443; 0,716)	0,250

EARLY AQUISITION (PET 10) TBR **>=1,6** SUV uptake in normal brain (VOI mean)

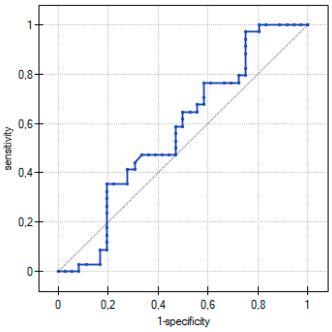

TBR brain	TBR60<=1,6	TBR60>/=1,6	Suma
True astrogliosis	15	22	37
True tumor	15	19	34
**Sum**	**30**	**41**	**71**

**sensitivity**	**specificity**	**PPV**	**NPV**	**AUC (95%CI)**	**p**
0,68	0,42	0,52	0,58	0,571 (0,434; 0,709)	0,304

Figure S2b

Differentiation of tumor from astrogliosis based on target-to-plexus ratio within FLAIR

STANDARD AQUISITION (PET 60) TBR > **1,00** SUV uptake in normal plexus (plexus ROI mean 60)

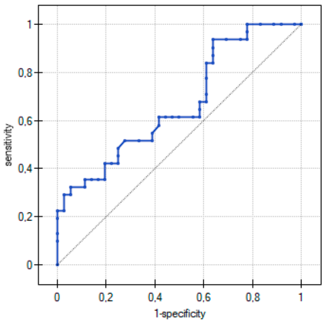

TBR plexus ROI mean	TBR60 plexus <=1,00	TBR60 plexus>1,00	Sum
True astrgliosis	13	23	36
True tumor	3	28	31
Sum	**16**	**51**	**67**

**sensitivity**	**specificity**	**PPV**	**NPV**	**AUC (95%CI)**	**p**
0,90	0,36	0,55	0,81	0,668 (0,538; 0,799)	0,018

EARLY AQUISITION (PET 10) TBR > **1,00** SUV uptake in normal plexus (plexus mean 10)

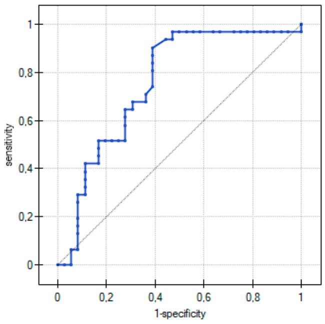

TBR plexus ROI mean	TBR10 plexus <=1,00	TBR10 plexus>1,00	Sum
True astrogliosis	22	14	36
True tumor	3	28	31
Sum	**25**	**42**	**67**

**sensitivity**	**specificity**	**PPV**	**NPV**	**AUC (95%CI)**	**p**
0,90	0,61	0,67	0,88	0,754 (0,633; 0,875)	<0,001

Figure S3. Comparison of TBR plexus (max values) with TBR plexus ROI mean (mean values).

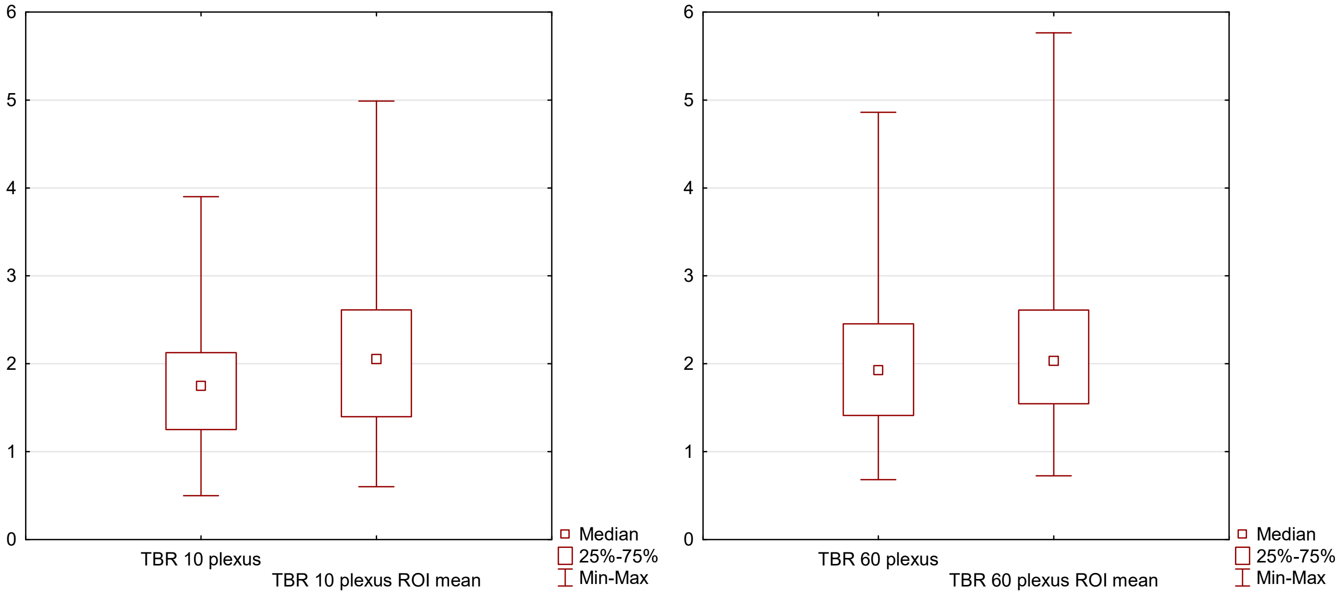

Figure S4. Comparison of ROC curves for TBR plexus in early and late acquisition, p<0.058.

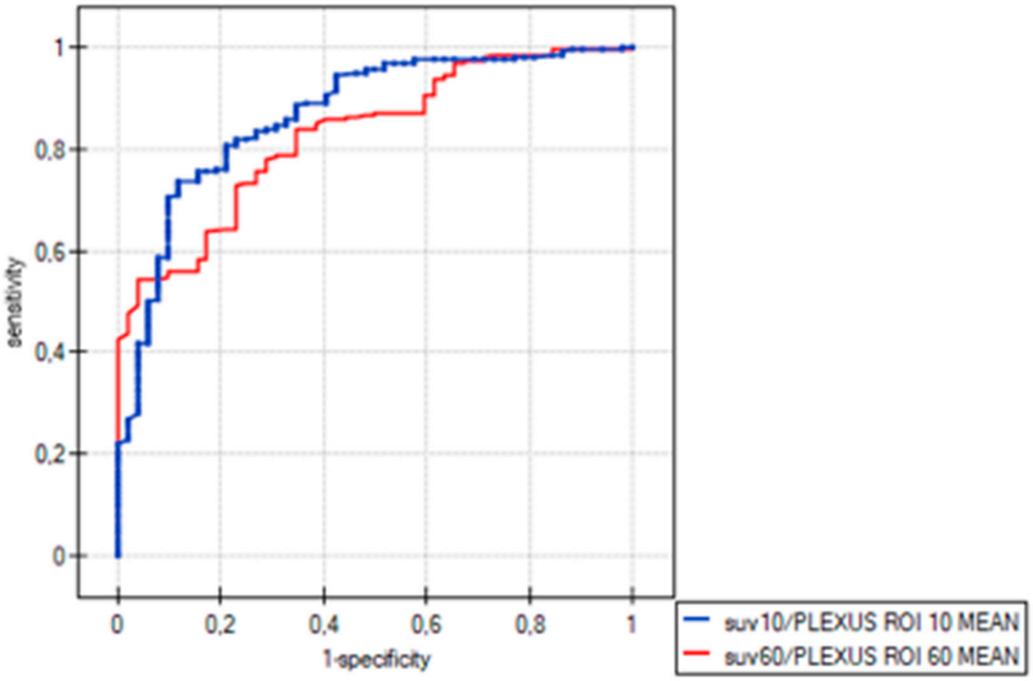

Figure S5. Comparison of values within structures defined with iPLAN and Syngovia.

Nr podkl.	Test HSD Tukeya; zmienna DV_1 (DO STATYSTYKI_1)<br>Przybliżone prawdopodobieństwa dla testów post hoc<br>Błąd: MS powt. pomiarów = .02575. df = 40.000			
	R1	1<br>1.1367	2<br>.97857	3<br>1.1686
1	SUV PLEXUS 10 (iPlan) MAX		0.007685	0.796744
2	PLEXUS ROI 10 MEAN (Syngovia)	0.007685		0.001333
3	PLEXUS ROI 10 MAX (Syngovia)	0.796744	0.001333	

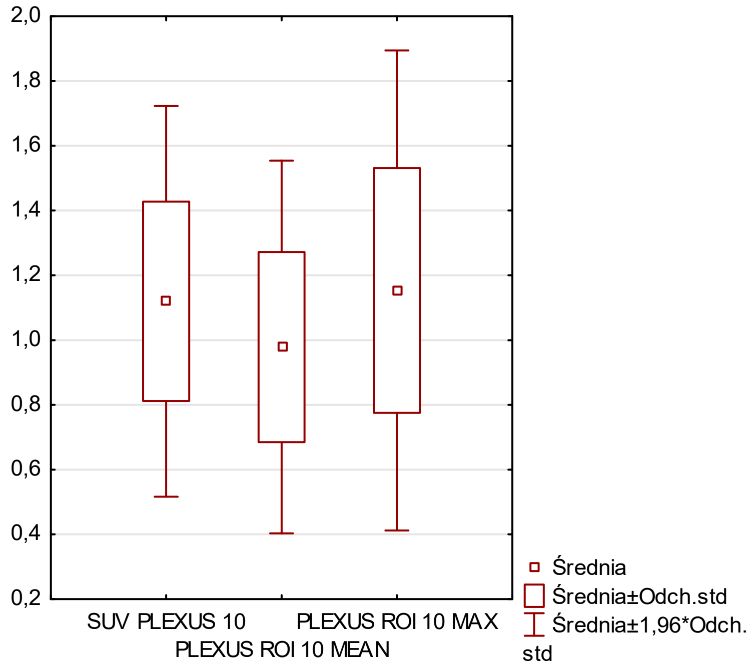

Nr podkl.	Test HSD Tukeya; zmienna DV_1 (DO STATYSTYKI_1)<br>Przybliżone prawdopodobieństwa dla testów post hoc<br>Błąd: MS powt. pomiarów = .01543. df = 40.000			
	R1	1<br>.98429	2<br>.91905	3<br>1.0700
1	SUV PLEXUS 60 MAX (iPlan)		0.217083	0.077374
2	PLEXUS ROI 60 MEAN (Syngovia)	0.217083		0.001019
3	PLEXUS ROI 60 MAX (Syngovia)	0.077374	0.001019	

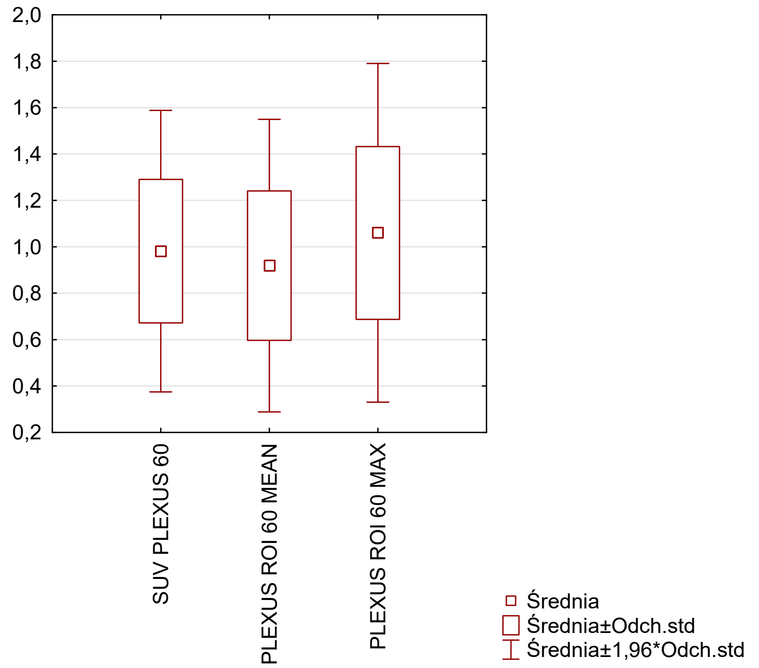

Timepoint 10	Test T dla prób zależnych (SUV dane pacjenci) Zaznaczone różnice są istotne z p < .05000				
	t	df	p	Ufność -95.000%	Ufność +95.000%
THALAMUS 10 (Syngovia)					
THALAMUS 10 (iPLAN)	-1.51939	12	0.154565	-0.159771	0.028489

	N	Mean	Median	Minimum	Maksimum
THALAMUS 10(Syngovia)	13	0.790769	0.770000	0.570000	1.020000
THALAMUS 10(iPLAN)	13	0.856410	0.773333	0.570000	1.400000

Timepoint 60	Test T dla prób zależnych (SUV dane pacjenci) Zaznaczone różnice są istotne z p < .05000				
	t	df	p	Ufność -95.000%	Ufność +95.000%
THALAMUS 60(Syngovia)					
THALAMUS 60 (iPLAN)	1.024733	12	0.325703	-0.011840	0.032865

	Nważnych	Średnia	Mediana	Minimum	Maksimum
THALAMUS 60 (Syngovia)	13	0.913846	0.970000	0.600000	1.080000
THALAMUS 60 (iPLAN)	13	0.903333	0.946667	0.600000	1.080000
